# Protocol: A simple phenol-based method for 96-well extraction of high quality RNA from Arabidopsis

**DOI:** 10.1186/1746-4811-7-7

**Published:** 2011-03-13

**Authors:** Mathew S Box, Vincent Coustham, Caroline Dean, Joshua S Mylne

**Affiliations:** 1 John Innes Centre, Norwich Research Park, Norwich, Norfolk, NR4 7UH, UK; 2 The University of Queensland, Institute for Molecular Bioscience, St Lucia, Queensland, Australia; 3 Current Address: Institut Jean-Pierre Bourgin, UMR1318 INRA-AgroParisTech, Bâtiment 7, INRA Centre de Versailles-Grignon, Route de St-Cyr (RD10), 78026 Versailles Cedex France

## Abstract

**Background:**

Many experiments in modern plant molecular biology require the processing of large numbers of samples for a variety of applications from mutant screens to the analysis of natural variants. A severe bottleneck to many such analyses is the acquisition of good yields of high quality RNA suitable for use in sensitive downstream applications such as real time quantitative reverse-transcription-polymerase chain reaction (real time qRT-PCR). Although several commercial kits are available for high-throughput RNA extraction in 96-well format, only one non-kit method has been described in the literature using the commercial reagent TRIZOL.

**Results:**

We describe an unusual phenomenon when using TRIZOL reagent with young Arabidopsis seedlings. This prompted us to develop a high-throughput RNA extraction protocol (HTP96) adapted from a well established phenol:chloroform-LiCl method (P:C-L) that is cheap, reliable and requires no specialist equipment. With this protocol 192 high quality RNA samples can be prepared in 96-well format in three hours (less than 1 minute per sample) with less than 1% loss of samples. We demonstrate that the RNA derived from this protocol is of high quality and suitable for use in real time qRT-PCR assays.

**Conclusion:**

The development of the HTP96 protocol has vastly increased our sample throughput, allowing us to fully exploit the large sample capacity of modern real time qRT-PCR thermocyclers, now commonplace in many labs, and develop an effective high-throughput gene expression platform. We propose that the HTP96 protocol will significantly benefit any plant scientist with the task of obtaining hundreds of high quality RNA extractions.

## Introduction

The scale of experiments conducted in modern plant molecular biology has grown such that hundreds or thousands of plant samples need to be processed by the researcher for use in a range of downstream applications, such as quantitative trait mapping, mutant screening and the analysis of gene expression in natural accessions-a rapidly growing resource for Arabidopsis research. Real time qRT-PCR is a common downstream application in such experiments and has become a major platform for high-throughput transcript profiling [1]. A significant bottleneck for many researchers is the acquisition of sufficient quantities of high quality RNA from such a large number of samples in a time and cost-effective manner. Although downstream technologies such as real time qRT-PCR have increased in their speed and capacity, the approaches to scale up the isolation of RNA have lagged behind.

Conventional RNA isolation techniques are based on a 1.5 mL micro-centrifuge tube format (or larger) using commercially available spin/vacuum-column kits or organic solvents such as TRIZOL (Invitrogen) and phenol. Although effective for low-throughput applications, isolating RNA from thousands of samples in micro-centrifuge tube format is incompatible with modern demands for high-throughput applications. A large number of protocols have been published for isolating high quality RNA however, only one high-throughput 96-well protocol is apparent in the published literature. This uses the commercial reagent TRIZOL (Invitrogen) for 96-well format nucleic acid extraction from Arabidopsis tissues, where it was favoured for its ability to simultaneously extract both DNA and RNA in a small number of steps [2]. However, some concerns have arisen regarding the suitability of TRIZOL for plant RNA isolation, for example, Bilgin *et al. *[3] showed that RNA extracts prepared with TRIZOL contained high levels of organic contaminants. We provide further evidence that adds to these concerns and explored an alternative to TRIZOL when developing our own high-throughput RNA extraction protocol to facilitate high-throughput transcript profiling.

Cheap, reliable and with a proven track record for a wide range of plant tissues and species, phenol provides an excellent alternative to TRIZOL. We developed a 96-well RNA extraction protocol by adapting a simple phenol:chloroform-LiCl method (P:C-L) [4,5] that we have used extensively [6-8]. We optimised the P:C-L protocol for 96-well racked collection tubes (1.2 mL) and greatly reduced the time required to complete the P:C-L method by removing the lengthy selective precipitation of RNA using LiCl [9,10]. Removal of the LiCl step has the added benefit of significantly increasing the representation of RNA species of small molecular weight [9] but results in unwanted gDNA. We elected a strategy that uses primers that bridge exons (as suggested in Czechowski *et al. *[11] and Gutierrez *et al.*[12]) to control the influence of contaminating gDNA in real time qRT-PCR, significantly reducing the time and cost of obtaining large numbers of RNA samples suitable for immediate use in real time qRT-PCR.

We demonstrate that phenol is more suitable than TRIZOL for high-throughput RNA extraction from a broad range of developmental stages. Our phenol-based high-throughput RNA extraction protocol (HTP96) can simultaneously isolate good yields of high quality RNA from 192 samples in less than three hours, with minimal loss or degradation of samples (typically <1%). The efficacy of the HTP96 protocol is demonstrated by presenting the results of simultaneous RNA extraction from a large number of different Arabidopsis accessions sampled at various developmental stages, from newly germinated seedlings to mature 30-day old plants. Electrophoresis and UV-spectrometry are used to demonstrate the quality and yield of RNA. The suitability of HTP96 RNA for sensitive downstream applications was established by real time qRT-PCR without additional purification, quantitation or DNase treatment.

To date we have used the HTP96 protocol to isolate total RNA from more than 3,000 Arabidopsis samples. The HTP96 RNA protocol has vastly improved our sample throughput, facilitating high-throughput screening of large numbers of transformants and transcript profiling in hundreds of Arabidopsis accessions. Although developed for Arabidopsis, we believe the HTP96 protocol will significantly benefit any plant scientist with the task of obtaining hundreds of high quality RNA extractions in a time and cost-effective manner.

## Materials and methods

### Consumables

NOTE: If using alternative plastic ware, make certain it is resistant to phenol and chloroform.

• 96-well racked 1.2 mL collection tubes and microtube caps (preferably in 8-tube strip format; QIAGEN, Cat.# 19560, #19566).

• (Optional) 1.5 mL micro-centrifuge tubes.

• (Optional) Powder funnels (Simport, Cat.# F490-4).

• 3 mm tungsten-carbide grinding beads (QIAGEN, Cat.# 69997). NOTE: 3 mm steel beads provide a cheaper alternative.

• 96-well PCR plates (ABgene, Cat.# AB-0700) and PCR seals (Bio-Rad, Cat.# MSB1001).

• Multi-channel pipette reagent reservoirs (60 mL).

### Reagents

• Liquid nitrogen and dry ice.

• RNA Extraction Buffer (RE buffer; 0.1 M Tris pH 8.0, 5 mM EDTA pH 8.0, 0.1 M NaCl, 0.5% SDS). Add 1% 2-mercaptoethanol before use.

• Acidified phenol pH 4.3 ± 0.2 (Sigma-Aldrich, Cat.# P4682).

• Chloroform.

• Isopropanol (2-propanol).

• 3 M sodium acetate (pH 5.2).

• 70% ethanol.

• Nuclease-free water.

• (Optional) DNase e.g. TURBO DNA-*free*™ (Ambion, Cat.# AM1907).

### Equipment

• Fume cupboard for handling phenol, chloroform and 2-mercaptoethanol.

• Bead-mill, preferably with a cryo-adapter capable of accommodating 96-well format collection tube racks and/or 1.5 mL micro-centrifuge tubes, e.g. GenoGrinder 2010 (SpexCertiprep) or similar.

• Plate mixer, e.g. the Vortex Genie 2 mixer fitted with a 96-well plate adapter (Scientific Industries, Inc.).

• Plate centrifuge at room temperature capable of speeds ≥4000 × g, (e.g. Sigma 4-15 C centrifuge fitted with a QIAGEN plate rotor-Nr.09100).

• Gel electrophoresis system.

• (Optional) Agilent 2100 Bioanalyzer (Agilent Technologies).

• NanoDrop ND-1000 Spectrophotometer (NanoDrop Technologies Inc.).

• Suitable multi-channel pipette (manual 8-channel 50-300 μL pipettor recommended). NOTE: Avoid using electronic pipettes for sensitive pipetting steps.

### Plant growth conditions

Arabidopsis seeds were sown on soil in plastic pots (7 cm × 7 cm) and stratified for three days in a cold room (CR, constant humidity 8 hours light/16 hours dark at 4°C). Seedlings were grown in a controlled environment growth room (CER, 12 hours light/12 hours dark at 23 °C). In some cases seeds were stratified for three days and the seedlings pre-grown for seven days and subject to cold treatment for up to six weeks in a CR. Sowings for different treatments were planned such that seedlings were sampled at equivalent developmental stages.

### Isolation of RNA using TRIZOL and phenol:chlorofom-LiCl (P:C-L)

Typically, RNA was isolated from 200 mg of ground Arabidopsis tissue powder prepared under liquid nitrogen. For TRIZOL, RNA was extracted from tissue powder according to the manufacturer's instructions and resupended in 50 μL of nuclease-free water. P:C-L RNA extraction was conducted using a scaled-down version of protocols previously described [4,5] and resupended in 50 μL of nuclease-free water.

### Northern blotting and hybridisation

10 μg RNA was loaded on a denaturing 1.2% agarose gel and transferred to Hybond™ N+ membranes (GE Amersham) by capillary transfer [9]. The RNA blot was probed with specific P^32^-dCTP *β-TUBULIN *(At1g20010) and *18S *rDNA probes prepared using Klenow fragment. Blots were exposed on phosphor screens (Kodak) and imaged with a Typhoon 9200 Variable Mode Imager (Amersham Biosciences).

### Agarose gel electrophoresis

For each RNA sample, an equal volume of RNA was prepared with a 2× denaturing RNA loading buffer (95% formamide, 0.025% SDS, 0.025% bromophenol blue). The RNA was denatured by heating to 70°C for ten minutes with the RNA loading buffer and then run on a 1.2% agarose gel as described by [9]. The ethidium bromide stained gel was visualised using a Typhoon 9200 Variable Mode Imager (Amersham Biosciences).

### Microfluidic gel electrophoresis

RNA was first of all treated with TURBO DNA-*free*™ DNase (Ambion) according to manufacturer's instructions. 4 μL of DNase treated RNA (300 ng μCL^-1^) were run on an Agilent 2100 Bioanalyzer microfluidic electrophoresis chip according to the manufacturer's instructions.

### Gene expression analysis

1 μg of total RNA preparation was used in a first strand cDNA synthesis reaction (10 μL final volume) using Superscript III and oligo(dT)20 (Invitrogen) following the manufacturer's instructions. After cDNA amplification, the 10 μL reaction was diluted with 60 μL of nuclease-free water, 5 μL of which was used in a 20 μL real time qRT-PCR reaction using SYBR Green Jumpstart Taq Ready Mix (Sigma-Aldrich, Cat.# S4438) and a Roche Lightcycler 480II instrument. Expression of the MADS-box transcription factor *FLOWERING LOCUS C *(*FLC*, At5g10140) was normalised to *UBIQUITIN CONJUGATING ENZYME1 *(*UBC*, At1g14400) using the comparative Cq (quantification cycle) method [13-16]. Primers were designed to bridge exons: 5'-AGC CAA GAA GAC CGA ACT CA-3' and 5'-TTT GTC CAG CAG GTG ACA TC-3' for *FLC*; 5'-CTG CGA CTC AGG GAA TCT TCT AA-3' and 5'-TTG TGC CAT TGA ATT GAA CCC-3' for *UBC*.

## Protocol

### TRIZOL is unsuitable for extraction of RNA from very young Arabidopsis tissues

In deciding to develop a high-throughput RNA extraction method appropriate for the isolation of RNA from hundreds of Arabidopsis samples, over a broad range of developmental stages, we first examined the suitability of TRIZOL, as it had been described in previous high-throughput nucleic acid extractions [2] where it was favoured for its ability to simultaneously extract both DNA and RNA in a small number of steps. Using TRIZOL we could always isolate high yields of total RNA from Arabidopsis, but found unusual mRNA expression patterns in northern blots prepared using RNA from plants at different stages of growth, specifically low or undetectable expression of several mRNAs in younger seedling stages.

**Figure 1:**
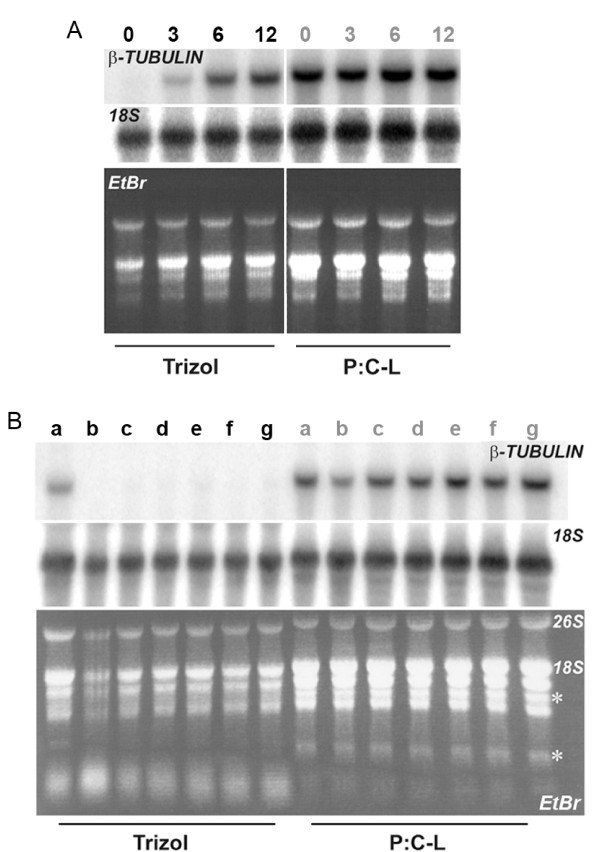
**TRIZOL is not suitable for extracting RNA from very young tissues**. RNA extracted using TRIZOL can satisfactorily isolate total RNA (*18S* bands) but very little mRNA (*β-TUBULIN* bands) is extracted from the very youngest Arabidopsis tissues. This bias is not evident in RNA prepared from the same tissue using P:C-L extraction. (A). Northern blot of TRIZOL and P:C-L-extracted RNA (10 μg) from samples grown for zero, three, six, or 12 days prior to a six week cold treatment and harvested in the cold. As the age of the seedlings increase, *β-TUBULIN* expression rises as a proportion of total RNA in the TRIZOL-extracted RNA preparations. By the time tissue was 12 days old, the bias observed in TRIZOL-extracted RNA had diminished. This effect is also observed for *FLC* (data not shown). (B). Northern blot comparing TRIZOL and P:C-L-extracted RNA (10 μg) of non cold treated five-day old (i.e. just germinated) Arabidopsis seedlings from several different genotypes (a-g) shows that the age-related bias observed in TRIZOL-extracted RNA is highly repeatable and not subject to growth conditions. TRIZOL and P:C-L extracted RNA preparations appear to differ significantly in the population of major RNA species visible by agarose gel electrophoresis (asterisks).

To explore this observation we extracted RNA from Arabidopsis seedlings grown at ambient temperature for three, six or twelve days followed by six weeks of cold treatment before immediately harvesting entire seedlings after the cold (Figure 1A). Northern analysis showed that as the age of the seedlings increased *β-TUBULIN *expression grew as a proportion of total RNA in the TRIZOL-extracted RNA (as indicated by *18S *rRNA expression). However, when an equal quantity of the same tissue was used for RNA extraction by P:C-L [3] no such bias in mRNA extraction was observed. This TRIZOL-specific phenomenon appeared to diminish as the age of the tissue used in the extraction increased. We explored this phenomenon further by harvesting five-day old (i.e. just germinated) Arabidopsis seedlings from a range of different accessions and mutants. These results confirmed an age-related bias in mRNA isolation from the very youngest Arabidopsis seedlings that was not evident when using P:C-L extraction.

Closer inspection of the RNA isolated from the youngest Arabidopsis seedlings using TRIZOL reveal several interesting differences when compared with RNA isolated from an equal quantity of the same tissue using P:C-L extraction. RNA yield did not vary greatly, UV-spectrometry indicated high yields of total RNA for both methods. From 200 mg of ground plant material the TRIZOL method isolated, on average, 52 ± 35 μg total RNA, whereas from the same starting tissue the P:C-L method isolated an average of 120 ± 32 μg total RNA. Inspection of TRIZOL and P:C-L extracted RNA by gel electrophoresis revealed several differences in the total RNA profile (Figure 1B). Firstly, TRIZOL appears to isolate more of the smallest RNAs than the P:C-L approach, most likely due to the absence of a LiCl precipitation step (LiCl is ineffective at precipitating small RNAs [9]). Secondly, TRIZOL failed to extract two classes of RNA whose bands are marked in the P:C-L RNA lanes with asterisks. Taken together these data suggest that the RNA population being isolated by the TRIZOL and P:C-L methods differ when using young (<12-day old) Arabidopsis seedlings.

These observations support our hypothesis that although TRIZOL can satisfactorily isolate high yields of total RNA, very little mRNA is extracted from younger Arabidopsis tissue. Despite repetition of these results in independent experiments we are unable to explain this phenomenon. A recent paper by Bilgin *et al. *[3] compared TRIZOL to other RNA extraction methods and found that RNA extracted by TRIZOL contained high levels of organic contamination as measured by the A_230/260 _ratio. The authors presented high-resolution microfluidic electrophoresis of soybean RNA extracted by several methods including TRIZOL, but there were no similar differences in the banding profile to what we asterisked in our gel image (Figure 1B). No mention was made of the stage of development the soybean tissue was at, which in light of our results, would be interesting as we found for Arabidopsis the phenomenon wanes in severity as tissue age increases.

The absence of this phenomenon when we used a P:C-L RNA extraction protocol (Figure 1) prompted us to use phenol as an alternative to TRIZOL in the development of a high-throughput RNA extraction protocol we call the HTP96 method, which we will now describe.

### The HTP96 RNA extraction protocol

#### Harvesting plant tissue

The HTP96 RNA extraction protocol has been developed for sampling tissues of all stages of Arabidopsis development including just germinated seedlings (three to five-days old) and mature plants. Whenever possible it is advisable to sample and homogenise tissue directly in 96-well format racked 1.2 mL collection tubes (hereafter *collection tubes*). However, if sampling more mature plants it may be convenient to collect and homogenise tissue using 1.5 mL micro-centrifuge tubes but this requires additional handling time. To obtain the best quality RNA it is essential that harvested material is frozen rapidly upon sampling and that the material is not allowed to thaw.

1. Prepare racks of 1.2 mL collection tubes (or 1.5 mL micro-centrifuge tubes) with 3 mm tungsten-carbide grinding beads (1 per sample).

2. On dry ice, harvest no more than 100 mg of fresh tissue into each collection tube. If using 1.5 mL micro-centrifuge tubes up to 200 mg of tissue can be collected and frozen immediately with liquid nitrogen. NOTE: Do not overfill tubes and ensure plant material is free of surface water for optimal tissue homogenisation.

#### Homogenisation of plant tissue (Timing: from 5 to 90 minutes)

3. Firmly secure 8-strip collection tube caps and allow them to cool at -80°C before homogenising the tissue. Samples are homogenised directly in collection tube racks using a commercial bead-mill. For most Arabidopsis tissues a fine powder can be produced in around 30 seconds on a moderate to high setting, we use a GenoGrinder 2010 at 1500 rpm for 30 seconds to prepare up to 384 samples simultaneously. NOTE: Avoid excessive homogenisation to prevent thawing of tissue powder.

4. (Optional). If using 1.5 mL micro-centrifuge tubes, homogenise samples in pre-cooled sample holders or specially manufactured cryo-adapters to avoid thawing. Powdered plant material must then be transferred (on dry ice) to pre-cooled 96-well racks of collection tubes. This must be done for each sample individually using liquid nitrogen cooled funnels. To prepare two duplicate plates in this way takes approximately 90 minutes (one minute per sample) providing samples are organized appropriately, we typically do this by sowing and collecting samples in a specified order to minimise excessive handling of fresh and frozen samples.

#### HTP96 RNA extraction (Timing: 60 minutes)

**Figure 2:**
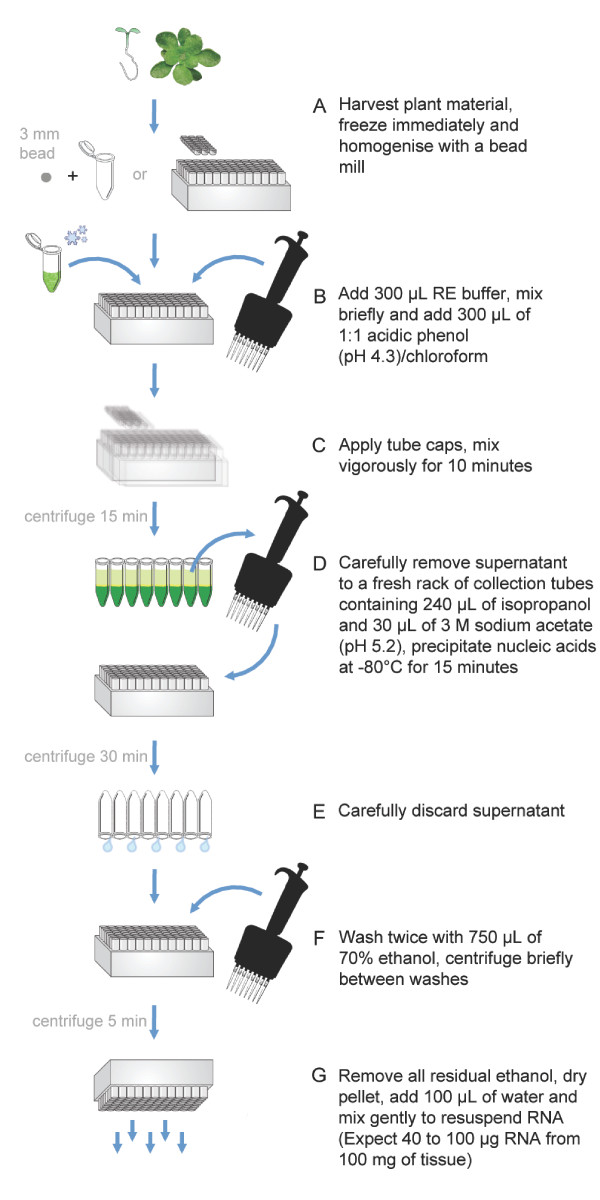
**Summary bench protocol for HTP96 RNA extraction**. High yields of good quality RNA are isolated from as little as 100 mg of fresh tissue using a streamlined P:C-L RNA extraction method in 96-well plate format. A. Harvested tissue is frozen immediately with liquid nitrogen and homogenised using a commercial bead mill. B. Add 300 μL RE buffer, apply collection tube caps and mix briefly. C. Briefly centrifuge to collect contents, add 300 μL of P:C (pH 4.3). Apply fresh collection tube caps and mix thoroughly. D. Nucleic acids in the supernatant are precipitated with 240 μL of isopropanol and 30 μL of 3 M sodium acetate (pH 5.2) at -80°C. E. Tape the collection tubes to the rack and discard the supernatant by inverting the collection tube rack. F. Wash the nucleic acid pellet twice with 70% ethanol. G. Remove all traces of ethanol and allow the nucleic acid pellet to dry before resuspending in nuclease-free water.

Typically two 96-well format racks of RNA are prepared simultaneously (192 samples) but this may be increased without significant difficulty if appropriate equipment is available. All steps of the protocol, including centrifugation, are carried out at room temperature. Use a fume hood and wear protective clothing and eyewear when handling 2-mercaptoethanol, phenol and chloroform. A bench summary of the HTP96 RNA extraction protocol is presented in Figure 2.

5. For 192 RNA extractions prepare 60 mL of RE buffer (add 600 μL of 2-mercaptoethanol to 60 mL RE buffer from a stock solution) and heat to 60°C in a waterbath. Prepare 60 mL of 1:1 acidic phenol:chloroform pH 4.3 ± 0.2 (P:C) and an excess (e.g. 1 L) of 70% ethanol. NOTE: In the absence of LiCl precipitation we use acidified phenol as it has previously been reported to reduce gDNA contamination [17].

6. Working carefully but quickly, add 300 μL of RE buffer to each collection tube of frozen tissue powder using a suitable multi-channel pipette. Firmly secure 8-strip collection tube caps and mix the contents vigorously using a plate mixer until the mixture thaws.

7. Briefly centrifuge each plate to collect the contents of each tube and prevent cross contamination during removal of collection tube caps. Add 300 μL of P:C to each collection tube, apply fresh tube caps and mix the contents vigorously for ten minutes.

8. Separate the aqueous (upper) and organic phases by centrifugation for 15 minutes at maximum speed (>4000 × g).

#### RNA precipitation (Timing: 60 minutes)

9. (During step 8) prepare fresh racks of collection tubes each containing 240 μL isopropanol and 30 μL 3 M sodium acetate (pH 5.2).

10. Carefully transfer up to 300 μL of the aqueous phase to the freshly prepared racks of collection tubes, apply a PCR plate seal and briefly mix using a plate mixer at low speed. NOTE: Remove the aqueous phase carefully; this is best done using a manual rather than electronic multi-channel pipettor, providing greater control over the pipetting action thereby avoiding disturbance of the interphase layer.

11. Precipitate the nucleic acids at -80°C for 15 minutes. If small amounts of tissue debris are transferred at this stage they are usually removed in the remaining steps.

12. Collect the nucleic acid precipitate by centrifugation for 30 minutes at maximum speed (>4000 × g). Do not be alarmed if the samples are frozen after precipitation at -80°C, they will thaw rapidly during centrifugation.

#### Washing RNA (Timing: 30 minutes)

13. Ensure all collection tube strips are intact, secure the collection tubes to the rack with masking tape and discard the supernatant by inverting the plate over a suitable container. As the nucleic acids are precipitated at this stage the possibility of cross-contamination is minimal (a pipette can be used if preferred). NOTE: If the 8-strip collection tube format is damaged, separated collection tubes can be lost during inversion of the tube rack. This is easily avoided by careful handling.

14. Wash the nucleic acid pellets with 600 μL of 70% ethanol to remove SDS, EDTA and other contaminants, apply a PCR plate seal, mix gently for a short time and centrifuge at maximum speed (>4000 × g) for five minutes. Discard the ethanol as described in step 13 and wash a second time.

15. Remove any remaining traces of ethanol by inverting the racked collection tubes onto absorbent paper (or by using a pipette) and allow the pellets to air dry. Drying is facilitated by periodically knocking the racks firmly, but gently, several times onto absorbent paper. The RNA pellet is considered dry when no further drops of liquid appear, this typically requires 30 minutes with three or four rounds of knocking onto absorbent paper. NOTE: Be careful not to lose the RNA pellets, they are securely attached to the tube but rough handling can dislodge them.

#### Dissolving RNA (Timing: 20 minutes)

16. Dissolve the nucleic acid pellet (mostly RNA) with 50-100 μL of nuclease-free water, apply a PCR plate seal and gently mix the contents using a plate mixer. Briefly centrifuge the dissolved RNA to remove any unwanted solid contaminants, e.g. tissue debris and transfer to a 96-well PCR plate. The samples can be stored at -20°C short term but should be stored long-term at -80°C. (Optional) At this stage aliquots of RNA may be treated with DNase as required. NOTE: In most cases DNase treatment is not necessary if HTP96 RNA is to be used for real time qRT-PCR.

## Comments

### Analysis of HTP96-extracted RNA quality by electrophoresis

**Figure 3:**
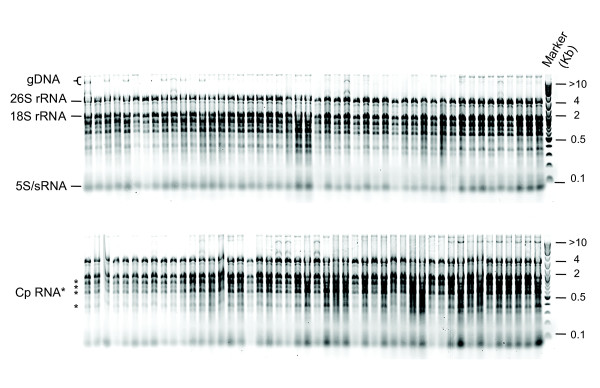
**Agarose gel electrophoresis of HTP96-extracted RNA**. Agarose (1.2% *w/v*) gel electrophoresis of RNA (~1 μg) isolated simultaneously from seven-day old seedlings of 96 Arabidopsis accessions using the HTP96 protocol. HTP96 RNA is highly intact as indicated by the clear cytosolic and plastidic (Cp, asterisks) ribosomal bands. RNA species of low molecular weight are also apparent (sRNA). gDNA contamination is visible when using a high resolution imaging system (e.g. Typhoon 9200 Variable Mode Imager).

**Figure 4:**
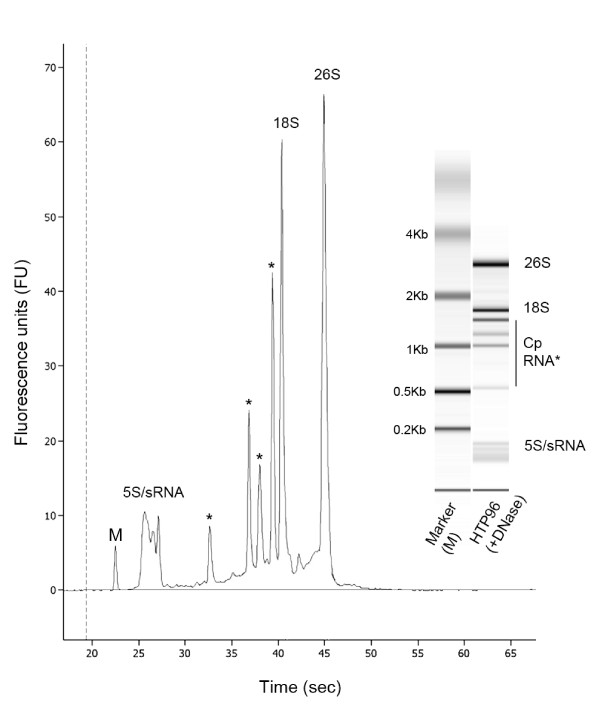
**Microfluidic electrophoresis of HTP96-extracted RNA**. The quality of HTP96 RNA (4 μL at 300 ng μL^-1^) was measured on an Agilent 2100 Bioanalyzer microfluidic electrophoresis chip following treatment with DNase. The microfluidic electrophoresis image (inset) and electropherogram are typical of high quality Arabidopsis RNA showing the clear cytosolic and plastidic (Cp, asterisks) ribosomal bands. RNA species of low molecular weight are also apparent. gDNA contamination is effectively removed by DNase treatment.

RNA quality was assessed using standard agarose gel electrophoresis (Figure 3) and Bioanalyzer microfluidic electrophoresis chips (Figure 4). Agarose gel electrophoresis shows that the HTP96 RNA protocol produces highly intact RNA as shown by the clear cytosolic and plastidic ribosomal RNA bands. Contamination with gDNA is not obvious when agarose gels are examined using a standard gel-doc system however, when using a high sensitivity device (e.g. the Typhoon 9200 Variable Mode Imager), moderate levels of gDNA contamination are evident in HTP96 RNA despite the use of acidified phenol. Microfluidic electrophoresis was carried out after treating HTP96 RNA with DNase. This confirms the high quality nature of HTP96 RNA and also demonstrates that gDNA contamination can of course be removed with DNase if required.

### Measuring HTP96 RNA concentration and purity by UV-spectrophotometry

**Figure 5:**
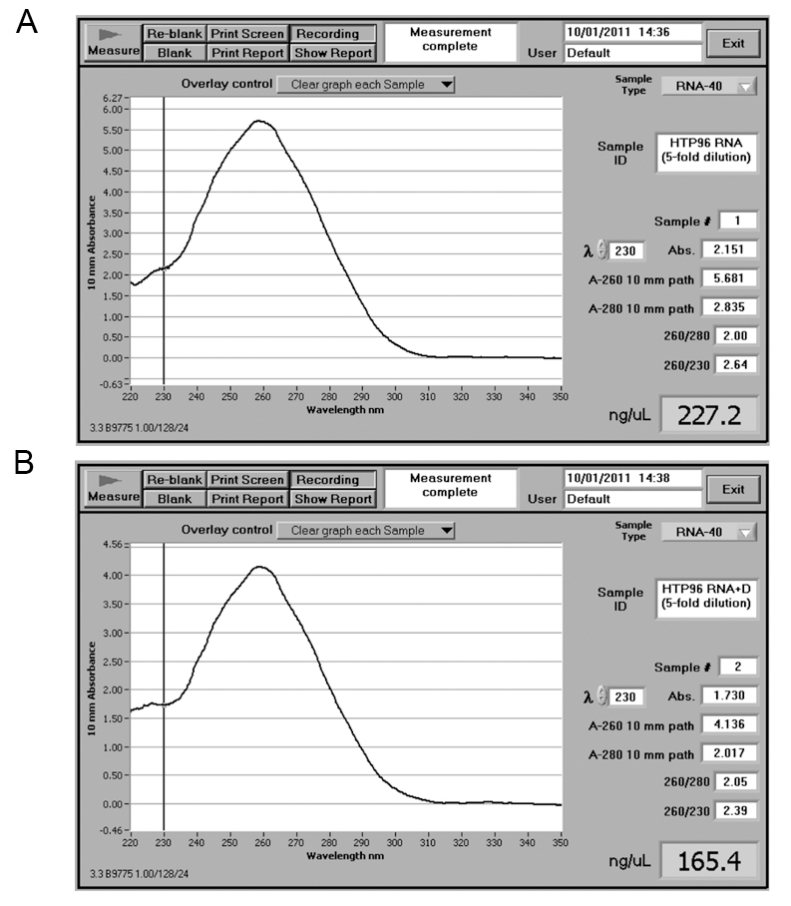
**Nanodrop spectrophotometry measurements of HTP96-extracted RNA**. HTP96 RNA extracts are of high quality and are free from appreciable levels of organic contaminants. **A**. HTP96 RNA measured immediately after extraction (five-fold dilution). **B**. HTP96 RNA measured after DNase treatment (five-fold dilution).

RNA concentration and purity can be determined spectrophotometrically [9] by measuring the absorbance at 230, 260 and 280 nm. RNA quantification is best done using a Nanodrop spectrophotometer with 1.5 to 2 μL of sample (Figure 5). For a typical HTP96 RNA preparation, measured after DNase treatment, the yield (as measured by A_260_) is in the region of 40 to 100 μg of RNA per 100 mg of starting tissue. The purity of the RNA is measured by calculating the ratio A_260_/A_280_, whereas the level of organic contaminants, e.g. polysaccharides and polyphenolics, is measured by the ratio A_260_/A_230_. Typically HTP96 RNA preparations have an A_260_/A_280 _ratio of ~2.0 and A_260_/A_230 _ratio >2.2 indicating that HTP96 RNA is suitable for immediate use in downstream applications without further purification.

### Analysis of HTP96-extracted RNA by real time qRT-PCR

The quality and uniformity of HTP96 RNA was assessed further by real time qRT-PCR. A common assay we use is to measure the effect of cold treatment on expression of the MADS-box transcription factor *FLOWERING LOCUS C *(*FLC*). Several Arabidopsis accessions with well characterised responses of *FLC *to cold treatment (e.g. Col *FRI*-*SF2 *[18] and Löv-1 [8]) were assessed. Equal quantities of the same tissue powder were used for RNA isolation using the HTP96 and the longer P:C-L extraction protocol that we have used previously [6-8] and which includes LiCl and DNase treatment. In contrast, HTP96 RNA was used immediately in reverse transcription and real time qRT-PCR without further purification or DNase treatment, instead we relied on a more time and cost-effective strategy to control against gDNA contamination using primers that bridge exons [11].

**Figure 6:**
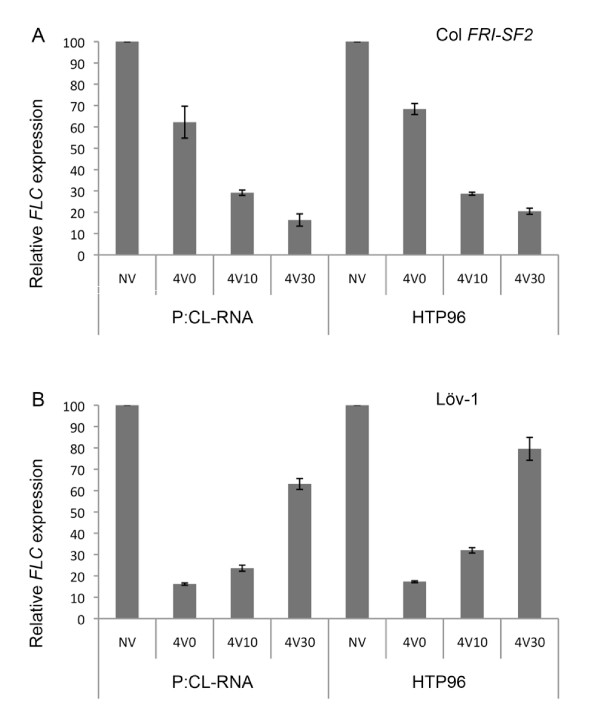
**Real time qRT-PCR analysis of *FLC *expression on plant samples harvested after four-weeks cold treatment and extracted using either the P:C-L or HTP96 methods**. The pattern of *FLC *expression was compared using RNA extracted from both the P:C-L and HTP96 methods. Plant samples from two different accessions were harvested without cold treatment (NV), and zero (4V0), ten (4V10) or 30 days (4V30) after a four-week cold treatment. Total RNA was extracted using either the P:C-L or HTP96 methods. Relative *FLC *expression of two biological replicates was assayed by SYBR green real time qRT-PCR, each consisting of three technical replicates. Error bars represent the standard error of the mean. **A**. *FLC *expression in Col FRI-SF2. **B**. *FLC *expression in Löv-1.

Previous expression analyses have shown that four-weeks cold treatment effectively silences *FLC *expression in the Col *FRI*-*SF2 *accession [18] but not in the Löv-1 accession [8]. We saw patterns of *FLC *expression consistent with this from both P:C-L and HTP96 extracted RNA (Figure 6). These data provide a convincing demonstration that the measures taken to adapt the standard P:C-L extraction protocol to 96-well format do not compromise the quality of RNA extracted from seedlings and mature plants. The similarity of data obtained from DNase treated P:C-L and non-DNase treated HTP96 RNA also indicates that the use of real time qRT-PCR primers designed to bridge exons controlled the moderate level of gDNA contamination present in HTP96 RNA preparations. This observation was further confirmed by melting curve analysis and real time qRT-PCR with HTP96 RNA minus reverse transcription (data not shown). Furthermore, the consistency of data between independent biological replicates demonstrates that the HTP96 protocol is able to isolate RNA in 96-well format without cross contamination.

**Figure 7:**
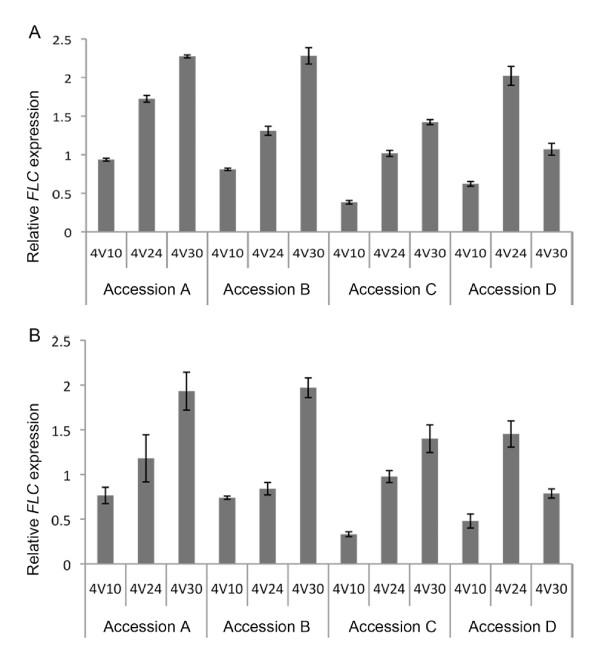
**Real time qRT-PCR analysis of *FLC *expression on plant samples harvested after four-weeks cold treatment and extracted using the HTP96 method**. Reverse transcription was carried out with and without RNA adjustment. Reverse transcription (RT) was simplified by using a defined volume of RNA preparation in each RT reaction. Plants samples of four different accessions were collected ten days (4V10), 24 days (4V24) or 30 days (4V30) after a four-week cold treatment. Total RNA was extracted using the HTP96 method. The reverse transcription reaction was performed using either 2 μL of RNA preparation, or by adjusting RNA concentration to 1 μg in a 10 μL final reaction volume. Relative *FLC *expression was assayed by SYBR green real time qRT-PCR using three technical replicates. Error bars represent the standard error of the mean. **A**. *FLC *expression assayed without prior RNA quantification. **B**. *FLC *expression assayed from 1 μg of total RNA.

To test the uniformity of RNA obtained using the HTP96 protocol we repeated the real time qRT-PCR analysis of *FLC *with four other Arabidopsis accessions. An equal quantity of tissue was harvested, homogenised and subject to RNA extraction using the HTP96 protocol as before. We tested uniformity by simplifying the reverse transcription step using a defined volume (2 μL) of RNA preparation without prior quantification and compared this to real time qRT-PCR results obtained with precisely quantified RNA (1 μg). Simplification of reverse transcription did not cause any significant bias in the linearity of mRNA amplification to cDNA as seen by real time qRT-PCR (Figure 7).

## Conclusion

Here we demonstrate concern over the use of TRIZOL for the extraction of RNA from the youngest Arabidopsis seedlings and suggest that phenol provides a cheaper and more reliable alternative. We describe an optimised, 96-well format high-throughput RNA extraction protocol (HTP96) based on a simple, cheap and reliable P:C-L method [4,5] that is widely used to isolate RNA from a broad variety of species and plant tissues. UV-spectrophotometry and microfluidic electrophoresis show that the HTP96 protocol is able to isolate good yields of highly intact RNA (40 to 100 μg per 100 mg of starting tissue), that is free from appreciable levels of contaminating proteins, phenol or salts (A_260_/A_280 _~2.0 and A_260_/A_230 _>2.2) and is of comparable quality to RNA isolated using a standard P:C-L extraction protocol but with improved throughput (typically, 192 samples in three hours).

We have shown that HTP96 RNA is suitable for immediate use in reverse transcription and real time qRT-PCR. Quantification of RNA can be omitted due to the high level of consistency in RNA yield and quality when using a similar input of tissue. Furthermore, we have shown that the use of exon-bridging real time qRT-PCR primers controlled the moderate gDNA contamination present in HTP96 RNA preparations. This removes the need for DNase treatment and LiCl precipitation, facilitating high-throughput analyses of gene expression. However, in cases where primers cannot be designed to bridge exons, e.g. when working with intronless transcripts (21.7% of Arabidopsis genes lack introns [19], or when real time qRT-PCR is not the intended application for HTP96 RNA, contaminating gDNA can be removed by DNase treatment.

The HTP96 protocol is a novel application of a well-established method of isolating high quality RNA. By adopting a 96-well format we have developed an improved protocol that allows phenol:chloroform extraction to be used to isolate large numbers of RNA samples using commonly available laboratory equipment. This method provides a cheap alternative to expensive 96-well format kits or effective, but low-throughput methods. We have since used the HTP96 protocol to assess gene expression in thousands of samples from hundreds of Arabidopsis accessions in conjunction with 96 and 384-well real time qRT-PCR thermocyclers, now commonplace in many laboratories.

We propose that the HTP96 protocol is of broad significance to the wider plant science community and will be fruitful for other users wishing to conduct high-throughput transcript profiling in a time and cost effective manner.

## Competing interests

The authors declare that they have no competing interests.

## Authors' contributions

JSM compared TRIZOL with P:C-L. MSB, VC and JSM developed the HTP96 extraction protocol. VC performed the gene expression analysis. MSB and JSM wrote the manuscript. All authors contributed to the preparation and approval of the final manuscript.
